# Multi-country comparison of delivery strategies for mass campaigns to achieve universal coverage with insecticide-treated nets: what works best?

**DOI:** 10.1186/s12936-016-1108-x

**Published:** 2016-02-03

**Authors:** Celine Zegers de Beyl, Hannah Koenker, Angela Acosta, Emmanuel Obi Onyefunafoa, Emmanuel Adegbe, Anna McCartney-Melstad, Richmond Ato Selby, Albert Kilian

**Affiliations:** Malaria Consortium, 56 Leonard St, London, EC2A 4LT UK; Johns Hopkins Center for Communication Programs, 111 Market Place, Baltimore, MD USA; Malaria Consortium, Abuja, Nigeria; Malaria Consortium, Kampala, Uganda; Tropical Health LLP, Montagut, Spain

**Keywords:** Malaria, Mass campaigns, Insecticide-treated nets, Delivery strategy

## Abstract

**Background:**

The use of insecticide-treated nets (ITNs) is widely recognized as one of the main interventions to prevent malaria. High ITN coverage is needed to reduce transmission. Mass distribution campaigns are the fastest way to rapidly scale up ITN coverage. However, the best strategy to distribute ITNs to ensure household coverage targets are met is still under debate. This paper presents results from 14 post-campaign surveys in five African countries to assess whether the campaign strategy used had any effect on distribution outcome.

**Methods:**

Data from 13,901 households and 14 campaigns from Ghana, Nigeria, Senegal, South Sudan and Uganda, were obtained through representative cross-sectional questionnaire surveys, conducted three to 16 months after ITN distribution. All evaluations used a multi-stage sampling approach and similar methods for data collection. Key outcomes examined were the proportion of households having received a net from the campaign and the proportion of households with one net for every two people.

**Results:**

Household registration rates proved to be the most important determinant of a household receiving any net from the campaign (adjusted odds ratio [OR] 74.8; 95 % confidence interval [CI]: 55.3–101.1) or had enough ITNs for all household members (adjusted OR 19.1; 95 % CI: 55.34–101.05). Factors that positively influenced registration were larger household size (adjusted OR 1.7; 95 % CI: 1.5–2.1) and families with children under five (adjusted OR 1.4; 95 % CI: 1.2–1.6). Urban residence was negatively associated with receipt of a net from the campaign (adjusted OR 0.73; 95 % CI: 0.58–0.92). Registration was equitable in most campaigns except for Uganda and South Sudan, where the poorest wealth quintiles were less likely to have been reached. After adjusting for other factors, delivery strategy (house-to-house vs. fixed point) and distribution approach (integrated versus stand-alone) did not show a systematic impact on registration or owning any ITN. Campaigns that used a universal coverage allocation strategy were more effective in increasing the proportion of households with enough ITNs than campaigns that used a fixed number of ITNs. Registering based on counting usual sleeping spaces resulted in higher levels of households with one net per two people among those receiving any campaign net (adjusted OR 1.6; 95 % CI: 1.07–2.48) than campaigns that registered based on the number of household members.

**Conclusion:**

All of the campaigns, irrespective of strategy, successfully increased ownership of at least one ITN. Delivery method and distribution approach were not associated with receipt of at least one ITN from the campaign. Rather, the key determining factor for receipt of at least one ITN from the campaign was a successful registration process, which depends on the ability of community volunteers to reach households during the exercise. Universal coverage campaigns, especially those that used a sleeping space allocation strategy, were more effective in increasing the proportion of households with enough ITNs. Maximizing registration completeness and using a universal coverage allocation strategy are therefore likely to improve campaign outcomes.

## Background

The burden of malaria remains unacceptably high in sub-Saharan Africa where 128 million people were infected at any one time in 2013 [1]. The use of insecticide-treated nets (ITNs) is one of the main interventions in preventing malaria. To reduce the overall malaria burden, nearly every at-risk individual must have access to an ITN. The World Health Organization recommends that countries strive for universal coverage (UC) using a combination of mass distribution complemented by continuous distribution through multiple channels such as antenatal and immunization services or primary schools [2].

Although mass distribution campaigns have proven to be the best approach to rapidly increase ITN coverage [3–19], the best strategy for implementing them is still under debate. Various strategies have been used to deliver, allocate, and distribute ITNs to households. For example, some campaigns used a fixed-point delivery strategy, whereas others distributed ITNs house-to-house, as in Ghana [20]. Some campaigns allocated a fixed number of nets per household (e.g., two nets per household, in Nigeria [21]) while others tailored the number of nets to be distributed according to household size (e.g., one net for every three people, in Madagascar) [22]. In Ghana and Senegal, ITNs were allocated on the basis of UC (i.e., one ITN for every two people or one ITN per sleeping space, respectively) [23, 24]. In Nigeria, some states chose to integrate the ITN campaign into child health activities (i.e., when oral polio, DPT, and measles immunizations were delivered or vitamin A was distributed to children under 5 years of age) [25]. In other places, ITNs were distributed through a stand-alone campaign.

Despite the volume of publications on mass ITN distribution campaigns, there has been no comparative analysis of the various approaches for implementing them. The malaria community needs to understand what works best in a variety of contexts to ensure the optimal use of resources for future campaigns. This paper presents results from 14 post-campaign surveys and attempts to assess whether the choice of campaign strategy had any effect on distribution outcomes and whether any other factors can be identified as determinants of successful campaigns.

## Methods

### Description of ITN campaigns

Data from 14 post-campaign household surveys conducted in Ghana, Nigeria, Senegal, South Sudan, and Uganda were used for the analysis (Table 1). The campaigns used a mix of delivery, allocation and distribution strategies. Most campaigns used a fixed-point delivery strategy where household members picked up ITNs at a central community distribution point such as a health clinic, while other campaigns delivered ITNs from house-to-house. Net allocation was either fixed (limited to two or three ITNs per household) or based on UC (henceforth called UC campaigns); UC campaigns either allocated one net for every two people in a given household or one net per sleeping space. Finally, campaigns were defined as having a stand-alone distribution strategy if only ITNs were delivered, while campaigns were defined as integrated if ITNs were distributed along with other services such as measles and polio vaccination.

**Table 1:** Characteristics of mass distribution campaigns

Campaign and year	Geographic coverage	Distribution strategy	Delivery strategy	Allocation strategy	Sample size target/achieved	Months between campaign and data collection^a^
Senegal, 2010–2011	Six regions^b^ in two phases	Stand-alone	Fixed point	UC (sleeping space)	1560/1540	3–12
Ghana, 2011–2012	Eastern region	Stand-alone	House-to-house	UC (sleeping space)	1020/1016	6
Kano State, Nigeria, 2009	Kano State in two waves	Stand-alone	Fixed point	Fixed (two ITNs per household)	1020/987	3–5
Niger State, Nigeria, 2009	Niger State	Stand-alone	Fixed point	Fixed (two ITNs per household)	1020/1001	6
Nasarawa State, Nigeria, 2010	Nasarawa State	Stand-alone	Fixed point	Fixed (two ITNs per household)	1020/1015	11
Ogun State, Nigeria, 2009	Ogun State	Stand-alone	Fixed point	Fixed (two ITNs per household)	1020/952	7
Anambra State, Nigeria, 2009	Anambra State	Stand-alone	Fixed point	Fixed (two ITNs per household)	1020/1012	4
Sokoto State, Nigeria, 2009	Sokoto State	Integrated with child health activities	Fixed point	Fixed (two ITNs per household)	1020/1008	6
Katsina State, Nigeria, 2010	Katsina State	Integrated with child health activities	Fixed point	Fixed (two ITNs per household)	1020/1017	6
Enugu State, Nigeria, 2011	Enugu State	Stand-alone	Fixed point	Fixed (two ITNs per household)	1020/1020	13
Lagos State, Nigeria, 2011	Lagos State	Stand-alone	Fixed point	Fixed (two ITNs per household)	1020/1020	8–9
Cross River State, Nigeria, 2011–2012	Cross River State in two waves plus urban (Calabar)	Stand-alone	Rural: House-to-house Urban: Fixed point	UC (top up by people)	1275/1254	4–16
South Sudan, 2008–2009	Northern Bahr-el Ghazal State^c^	Stand-alone	Fixed point	Fixed (by household size, maximum three ITNs)	510/510	6–10
Uganda, 2009–2010	Four districts in Western region^d^	Stand-alone	Fixed point	UC (sleeping space/people)^e^	600/549	5–9

*ITN* insecticide-treated net, *UC* universal coverage

^a^Ranges of time indicate single surveys that measured multiple phases of the same campaign

^b^Kaolack, Kaffrine, Sedhiou, Tambacounda, Kolda, Kedougou

^c^Counties: Aweil North, Aweil West, Aweil Centre

^d^Buliisa, Hoima, Kyankwanzi, Kiboga

^e^Depending on administrative district

Registration in each campaign consisted of door-to-door visits. The steps in registration and receipt of a net were as follows: (1) volunteers visited a household, counted the number of eligible beneficiaries, and assigned a number of nets based on an allocation strategy; (2) volunteers then issued a coupon; household members redeemed the coupon for the allocated number of nets. Census information was not used because it was often inaccurate and out of date. These campaigns were followed up 6–12 months later with an evaluation.

### Study design and sampling strategy

Each 6- to 12-month post-campaign evaluation consisted of a similar, cross-sectional household interview survey with a two-stage cluster sampling design, which allowed easy comparison of all surveys. The aim of the sampling strategy was to obtain a representative sample of the state or regional population, allowing inclusion of any village or household even if it had not been included in the campaign. A multi-stage sampling approach was used in which clusters (defined as villages) were selected at the first stage through the probability-proportionate-to-size method. In the second stage, a simple random sample of households was taken. A list of village households was used to randomly select an equal number of households in each cluster. The sample size and precision were calculated through a standard formula considering statistical parameters such as confidence interval (95 %), power (80 %), design effect (1.75), non-response (10 %), and average household size.

### Outcomes of interest

The main outcomes assessed in this study were the proportion of households that received at least one ITN from the campaign (also referred to as reach), and the proportion of households with enough nets (defined as having at least one ITN for every two people). Secondary outcomes included registration rates (the proportion of households that were registered by the campaign) and equity in registration or UC (the degree to which poorer quintiles were registered or had enough nets compared with wealthier quintiles).

### Data collection

Data collection methods were similar for all 14 surveys, allowing valid comparisons across region and country. A standard questionnaire was used, and each was tailored to the context where it would be used, but these adaptations were modest. Questions related to the key indicators presented in this paper were not modified. Survey teams had to approach people in the local language or dialect, which often differed among and within nations. Whereas the challenge of multiple languages cannot be entirely overcome, it was carefully anticipated. The training of field teams purposely included a session on key terms to ensure all interviewers understood the question and agreed on the wording in each language. This guaranteed consistency across teams and thus across surveys to the extent possible.

### Data entry, processing, and statistical analysis

All data were double-entered using EpiData software version 3.1. Both datasets were compared and any discrepant records were verified from the original questionnaires. Once this first stage of cleaning was finished, the dataset was transferred to Stata version 10.1 (StataCorp, College Station, TX, USA) for further consistency checks and preparation for analysis. The final analysis of pooled survey data included 13,901 households. Statistical analysis used contingency tables and Chi squared test for univariable analysis, and multivariable logistic regression modeling to assess associations between background characteristic and outcome variables of interest. Regression models were built using a stepwise inclusion approach with a p < 0.2 cut-off level for significance. The variable identifying the individual surveys was included in all models irrespective of significance in order to account for the data structure. Likelihood ratio tests were used to compare fit of nested models. All analyses accounted for sampling weights and any potential clustering effect using the survey family commands in Stata. The wealth index was computed at the household level using principal component analysis (PCA). The variables for household amenities, assets, livestock, and other characteristics that are related to a household’s socio-economic status were used for the computation. All variables were dichotomized, except those of animal ownership, where the total number owned was used. The first component of the PCA was used as the wealth index. Households were then classified according to their index value into quintiles. Quintiles were calculated separately for urban and rural strata to adjust for socio-economic differences. For analysis of individual members of the household or nets, the quintile allocation of the household was applied. A concentration index was used to analyze outcome differences by wealth. Standard errors and confidence intervals for the concentration indices were calculated using the formula suggested by Kakwani et al. [26]. Once all survey data were available, further consistency checks were performed to ensure all variable names and format and coding options were consistent among surveys. Finally, data were merged to obtain the database that was used for analysis.

### Ethical consideration

Ethical approval was sought for all evaluations from the appropriate ethics committee in each country. Before administering the questionnaire, the interviewer carefully read the information sheet and consent form to the respondent. The consent forms contained information on the objectives of the survey, the risks, benefits, and freedom of participation, and information on confidentiality plus interviewee rights.

## Results

### Household ITN ownership

The outcomes of each campaign are presented in Table 2. The proportion of households that had at least one ITN was generally low, considering that all the campaigns sought to reach every household. Less than a handful achieved rates of ownership of at least one ITN above 85 % (Ghana, Senegal, Uganda, and South Sudan) and four campaigns reached fewer than 60 % of households [Niger, Ogun, Lagos, and Cross River (urban) States in Nigeria].

**Table 2:** Outcome of the campaigns with respect to ITN ownership coverage, by delivery and allocation strategy (N = 13,901)

Location and allocation strategy	% of HH with any net before campaign (95 % CI)	% of HH with any ITN on survey day (95 % CI)	% of HH with one ITN per two people (95 % CI)	% of population with access to an ITN within HH (95 % CI)
Fixed-point delivery, fixed allocation (two ITNs per household)				
Kano State, Nigeria	13.2 (6.0–26.6)	69.3 (58.1–78.6)	30.1 (23.4–37.9)	43.9 (35.8–52.3)
Niger State, Nigeria	0.7 (0.3–1.7)	51.7 (40.3–62.9)	16.6 (12.4–21.8)	34.2 (25.9–43.7)
Nasarawa State, Nigeria	13.8 (9.8–19.2)	62.5 (55.1–69.5)	24.8 (20.3–29.9)	41.5 (35.8–47.5)
Ogun State, Nigeria	4.6 (2.7–7.6)	52.6 (42.0–62.9)	22.6 (15.9–30.7)	37.0 (28.2–46.7)
Anambra State, Nigeria	7.6 (5.2–10.8)	64.4 (56.9–71.3)	36.6 (31.0–42.6)	50.0 (43.2–56.8)
Sokoto State, Nigeria	7.2 (4.6–11.3)	64.0 (55.5–71.7)	30.6 (24.7–37.3)	49.0 (42.2–55.8)
Katsina State, Nigeria	3.5 (2.1–5.6)	73.8 (63.2–82.1)	36.5 (30.0–43.6)	56.2 (48.5–63.7)
Enugu State, Nigeria	4.1 (2.8–6.1)	72.3 (63.2–75.3)	35.3 (31.9–38.9)	54.6 (51.4–57.7)
Lagos State, Nigeria	5.9 (3.3–10.4)	46.3 (41.2–51.5)	17.8 (15.2–20.7)	32.2 (32.6–40.1)
Fixed-point delivery, fixed allocation (by household size, maximum three ITNs)				
South Sudan	1.7 (0.8–3.6)	87.7 (76.6–93.9)	44.1 (36.2–52.4)	No data^a^
Fixed-point delivery, universal coverage allocation (one ITN per two persons or one ITN per sleeping space)				
Senegal	39.9 (35.0–45.1)	93.9 (90.3–96.2)	42.3 (37.0–47.8)	75.2 (69.7–80.1)
Uganda	39.8 (33.0–46.9)	91.7 (83.9–95.9)	69.9 (63.2–76.0)	81.3 (74.1–86.9)
House-to-house delivery, universal coverage allocation (one ITN per two persons or one ITN per sleeping space)				
Ghana	12.0 (9.5–15.2)	91.2 (88.3–93.5)	51.2 (47.1–55.3)	74.5 (71.1–77.6)
Cross River State, Nigeria	11.3 (8.0–15.9)	61.6 (57.2–65.8)	24.2 (20.4–28.4)	45.9 (41.8–50.1)

*CI* confidence interval, *HH* household, *ITN* insecticide-treated net

^a^ No household roster data were collected

However, all campaigns increased household ITN ownership, and these gains were statistically significant for all locations. The largest increase in ownership of at least one ITN was in Ghana, where the campaign achieved a 79.2 % point increase. The campaign in Lagos, Nigeria had the lowest impact on household ownership with a difference of 40.4 % points. Overall, ownership of at least one ITN ranged from 46.3 % in Lagos to 93.9 % in Senegal, while the proportion of households with one ITN for two people (enough ITNs) ranged from 16.6 % in Niger State, Nigeria to 69.9 % in Uganda and population access to an ITN ranged from 32.2 % in Lagos to 81.3 % in Uganda.

### Registration and socio-economic determinants of household ITN ownership

**Fig. 1:**
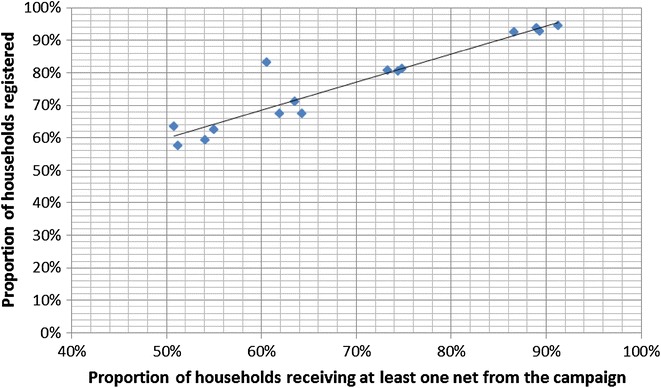
Association between household registration and receipt of at least one ITN from the campaign

Receipt of at least one ITN from the campaign was very closely associated with increasing household registration rates (Fig. 1), where a linear trend between registration rates and receiving at least one ITN from the campaign is observed (regression coefficient 0.86; p < 0.0001, R-squared 0.88). This implies that overall 86 % of those registered also received a campaign net. The campaign in Nasarawa State, Nigeria, had the most pronounced deviation from this trend. The proportion of households that received an ITN from the campaign once they were registered was only 69.8 %. This is likely due to an insufficient quantity of net coupons available for redemption, as only 66 % of those registered received a coupon.

Table 3 presents results from a logistic regression analysis looking at household characteristics and campaign registration, receiving any ITN from the campaign, and having enough ITNs. Results show that registration was the main predictor of whether a household received any ITNs (adjusted odds ratio [OR] 74.8; 95 % confidence interval [CI]: 55.34–101.05) or had enough ITNs (adjusted OR 19.1; 95 % CI: 14.31–19.50).

**Table 3:** Multi-variable logistic regression models of determinants of household registration, ownership of at least one ITN from the campaign, and having at least one ITN per two people (N = 13,901)

Factors of association^a^	Adjusted OR	95 % CI	P value
Outcome: Household registered by campaign			
Household of four people or more (vs three or less)	1.74	1.47–2.07	<0.001
Household with any child under five (vs no child under five)	1.37	1.20–1.58	<0.001
Household in urban area (vs. rural)	0.73	0.58–0.92	0.008
Poorest households (quintile 1 vs 2–5)	0.90	0.76–1.06	0.200
House-to-house delivery (vs fixed point)	0.73	0.48–1.12	0.149
UC allocation (vs fixed allocation)	0.76	0.54–1.07	0.112
Outcome: Household received any net from campaign			
Registered by campaign	74.79	55.34–101.05	<0.001
Household of four people or more (vs three or less)	1.28	1.06–1.55	0.010
Household with any child under five (vs no child under five)	0.76	0.64–0.89	0.001
Household in urban area (vs rural)	0.60	0.44–0.80	0.001
Outcome: Household has one ITN per two persons (sufficient ITNs)			
Registered by campaign	19.09	14.31–19.50	<0.001
Household of four people or more (vs three or less)	0.16	0.14–0.20	<0.001
Household with any child under five (vs no child under five)	0.62	0.55–0.69	<0.001
Household in urban area (vs rural)	0.74	0.63–0.87	<0.001

*CI* confidence interval, *ITN* insecticide-treated net, *OR* odds ratio

^a^All models included the survey to reflect the structure of the data (results not shown)

Households with four or more members were more likely to get registered (adjusted OR 1.74; 95 % CI: 1.47–2.07) and more likely to receive a campaign net if registered (adjusted OR 1.28; 95 % CI: 1.06–1.55), but less likely to receive enough ITN for all members (adjusted OR 0.16; 95 % CI: 0.14–0.20). Households with any children under five were also more likely to be registered (adjusted OR 1.37; 95 % CI: 1.20–1.58) and this was consistent across all countries. When the likelihood of receiving any ITN from the campaign was adjusted for registration, households with young children were slightly disadvantaged (adjusted OR 0.76). However, this relationship was driven by results from a few states in Nigeria (Lagos, Enugu, Anambra, and Kano). This implies that once registered, families with young children in these states were less likely to receive a campaign net compared to registered families of similar size; in all other Nigeria states and countries there was either no association or a positive association. Urban residence was consistently negatively associated with all three campaign outcomes. Poorer households were slightly less likely to be registered, but socio-economic status played no role in the other campaign outcomes.

### Delivery strategy, allocation strategy and household ownership

Table 2 organized campaign outcomes by delivery strategy (house-to-house vs. fixed point) and allocation strategy (fixed allocation vs. UC). Household ownership result by delivery strategy appeared mixed (both high and low). Table 2 also shows that campaigns that used a fixed allocation strategy of two or three ITNs per household (Nigeria and South Sudan) had lower proportions of households with one ITN for two people and lower levels of population access compared to campaigns that used a UC allocation strategy (Ghana, Senegal, and Uganda). The only exception was Cross River State in Nigeria, which was originally intended to be a UC top-up campaign. However, due to a shortage of nets, the number of nets distributed per household was capped at four. Inclusion of the delivery and allocation variables did not result in statistically significant or consistent associations in the logistic regression model for registration and were outside the inclusion criteria in the models for any ITN received and one ITN for two people (Table 3).

#### Among UC strategies, sleeping space vs. number of household members

Among campaigns that adopted a UC allocation strategy, some used the number of people to determine the number of nets a household would receive, while others used the number of sleeping places. Table 4 shows which of these two allocation strategies was associated with the likelihood of having enough ITNs. This was explored by logistic regression models using only campaigns with UC allocation and included only households that had received at least one net from the campaign (N = 3458). In Uganda, the two approaches had been used in the same campaign allowing for a direct comparison (N = 491). Results showed a consistent positive association between sleeping place allocation and enough ITNs in the household (adjusted OR 1.63; 95 % CI: 1.07–2.48).

**Table 4:** Predicting factors of a household with enough ITNs (one net per two people) on survey day among those that received at least one net from a universal coverage campaign

Factors of association^a^	All (n = 3458)			Uganda (n = 491)		
	Adjusted OR	95 % CI	P value	Adjusted OR	95 % CI	P value
UC allocation by sleeping place (vs by people)	1.63	1.07–2.48	0.025	1.60	1.02–2.52	0.042
Wealth quintile						
Lowest	1.00		<0.001	1.00		0.172
Second	1.09	0.78–1.53		1.21	0.59–2.46	
Middle	1.39	1.01–1.91		1.40	0.72–2.71	
Fourth	1.66	1.19–2.31		1.48	0.76–2.91	
Highest	1.91	1.37–2.64		2.79	1.20–6.53	
Household of four people or more (vs three or less)	0.25	0.18–0.35	<0.001	0.42	0.24–0.76	0.005
Household with any child under five (vs no child under five)	0.37	0.30–0.47	<0.001	0.20	0.10–0.38	<0.001

*CI* confidence interval, *ITN* insecticide-treated net, *OR* odds ratio, *UC* universal coverage

^a^All models included the survey to reflect the structure of the data (results not shown)

**Fig. 2:**
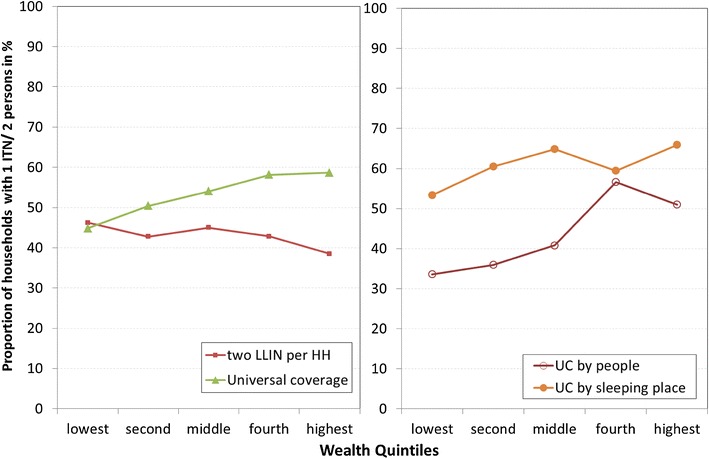
Relationship between allocation strategy, wealth quintile, and the proportion of households with sufficient ITNs if any nets were received from the campaign

Among households that had received at least one net from the campaign, those with four or more members (adjusted OR 0.25; 95 % CI: 0.18–0.35) and those having children under five (adjusted OR 0.37; 95 % CI: 0.30–0.47) were less likely to have enough ITNs. Upon closer examination, small households (three or fewer people) with children under five were much less likely to have enough ITNs compared to other small households without young children (51.3 vs. 85.3 %; adjusted OR 0.14; CI: 7.5–25.7), while this effect was only moderate in larger households (40.1 vs. 48.2 %; adjusted OR 0.46; CI: 34.8–61.5). This relationship was consistent in all countries with UC allocation in the sample. Other variables such as urban vs. rural residence, education of head of household, or time between the survey and campaign did not show a significant impact on households having one ITN for two people in the regression model. Households in the wealthiest quintile were more likely to have enough ITNs and this was explored further in the equity analysis presented in Fig. 2.

#### Allocation strategy and equity in household ownership

Access to any ITN through the campaign as well as having one ITN per two people was generally equitable across all campaigns. Between types of campaigns, however, some variation was observed. Figure 2 (left panel) compares ownership of one ITN per two people (enough ITNs) by wealth quintile between campaigns that use a UC and fixed number allocation. Among households who had obtained a net from a UC campaign, those from wealthier quintiles appear to slightly more likely to have enough ITNs. The concentration index for UC allocation was slightly positive, indicating that it was slightly “pro-rich” with 0.034 (95 % CI 0.015–0.053) while for fixed number allocation it was slightly negative, indicating that it was slightly “pro-poor” with −0.023 (−0.04–0.041).

A closer examination revealed that the type of UC allocation strategy affected equity in ownership of enough ITNs (Fig. 2, right panel). The concentration index of 0.013 (−0.008–0.034) for allocation by sleeping place was, in fact, very close to zero and thus very equitable, while the index for allocation by number of people was much less equitable, with a concentration index of 0.105 (0.065–0.146).

#### Allocation strategy and efficiency

Since UC allocation by sleeping place appeared to result in better and more equitable results for households having enough ITNs (if they were served by the campaign), the final question was whether type of UC allocation strategy was also associated with efficiency in supplying the right amount of nets.

Table 5 shows that allocation was not associated with efficiency; the level of oversupply varied dramatically within both allocation approaches, ranging from 4 to 30 % among campaigns that used sleeping space allocation and 8–25 % among campaigns that allocated based on number of people in the household. Even within Uganda, which had used both types of UC allocation strategies, no significant difference by allocation strategy was observed (p = 0.28).

**Table 5:** Proportion of households from UC campaigns undersupplied or oversupplied, by allocation strategy

	Undersupply (%)			Just right (%)	Oversupply (%)
	<1 ITN/3 persons	≥1 ITN/3 persons	Total undersupply	≥1 ITN/2 persons	≥1 ITN/person
UC by sleeping place					
Senegal	20.5	33.4	53.9	42.6	3.5
Ghana	17.2	26.5	43.7	42.9	13.4
Uganda	6.5	13.2	19.7	50.2	30.1
*Total*	*14.9*	*27.6*	*42.5*	*44.5*	*16.9*
UC by people					
Uganda	6.7	21.0	27.7	47.2	25.1
Cross River State, Nigeria	31.3	29.5	60.8	31.1	8.1
*Total*	*25.7*	*27.6*	*53.3*	*34.7*	*11.9*

*ITN* insecticide-treated net, *UC* universal coverage

### Registration

#### Registration rates and delivery strategy

Because registration appeared to be the primary determinant of household ownership, this element of the campaign process was explored further by looking at registration rates at the community level, with each survey cluster corresponding to a community. The 14 surveys included 795 communities. Registration rates were high in Senegal, Uganda, Ghana, and South Sudan with an average 92.6 % (95 % CI: 89.3–95.9) of sampled households within a community being registered. Only 8.3 % of the communities in these surveys had registration rates between 50 and 80 % while the remaining 91.7 % of communities had registration rates between 80 and 100 %. In the Nigeria surveys, on the other hand registration rates were very low. Only 43.4 % of communities had registration rates at 80 % or more; 41.8 % had between 50 and 79 %; 12.2 % had between 10 and 49 % and 2.6 % had less than 10 %.

#### Delivery strategy, allocation strategy and registration rates

When registration rates were grouped by delivery strategy (house-to-house *vs* fixed point) and allocation strategy (fixed allocation *vs* universal coverage), results were varied, with some campaigns having higher registration coverage and other campaigns having lower registration coverage regardless of delivery and allocation strategy (Table 6). There was no significant difference in registration rates by delivery approach (house-to-house *vs* fixed point); this was true overall and when Nigeria surveys were excluded (p > 0.05). Accordingly, inclusion of the delivery and allocation variables did not result in statistically significant or consistent associations in the logistic regression model for registration.

**Table 6:** Registration outcomes by delivery and allocation strategy (N = 13,901)

Location, delivery, and allocation strategy	% of households registered (95 % CI)	*N*	Among households registered	
			% receiving a coupon (95 % CI)	% receiving a least one ITN (95 % CI)
Fixed-point delivery, fixed allocation (two or three ITNs per household)				
Kano State, Nigeria	71.0 (61.7–78.8)	691	99.5 (98.3–99.8)	89.4 (82.4–93.8)
Niger State, Nigeria	57.5 (54.9–68.3)	645	94.9 (90.6–97.3)	88.9 (83.1–93.0)
Nasarawa State, Nigeria	83.2 (79.2–86.6)	724	66.3 (56.0–75.1)	69.8 (59.9–78.2)
Ogun State, Nigeria	62.6 (54.3–70.2)	496	96.0 (92.1–98.0)	88.0 (80.3–92.9)
Anambra State, Nigeria	80.5 (73.8–85.9)	830	96.3 (92.3–98.3)	92.3 (87.5–95.4)
Sokoto State, Nigeria	67.3 (58.2–75.3)	680	96.4 (93.3–98.1)	91.8 (87.6–94.7)
Katsina State, Nigeria	80.8 (72.5–87.0)	831	90.2 (81.4–95.1)	90.8 (81.9–95.6)
Enugu State, Nigeria	81.2 (77.3–84.6)	737	87.8 (84.6–90.4)	91.5 (87.4–94.4)
Lagos State, Nigeria	63.6 (55.7–70.9)	664	84.1 (78.2–88.7)	79.0 (72.5–84.3)
South Sudan	92.6 (88.2–95.4)	469	No data^a^	93.5 (90.3–95.7)
Fixed-point delivery, universal coverage allocation (one ITN per two persons or one ITN per sleeping space)				
Cross River State (urban), Nigeria	59.3 (52.0–66.3)	152	87.1 (65.2–96.1)	91.2 (86.4–94.3)
Senegal	93.7 (90.9–95.7)	1367	84.0 (78.5–88.4)	94.8 (92.1–96.7)
Uganda	92.8 (85.3–96.6)	510	78.2 (72.4–83.1)	96.3 (91.7–98.4)
House-to-house delivery, universal coverage allocation (one ITN per two persons or one ITN per sleeping space)				
Ghana	94.5 (92.4–96.0)	955	n.a.	96.6 (94.2–98.1)
Cross River State (rural), Nigeria	67.5 (62.9–71.8)	1102	n.a.	95.2 (92.0–97.3)

*CI* confidence interval, *ITN* insecticide-treated net

^a^Data on registration outcome were not collected during the evaluation

**Fig. 3:**
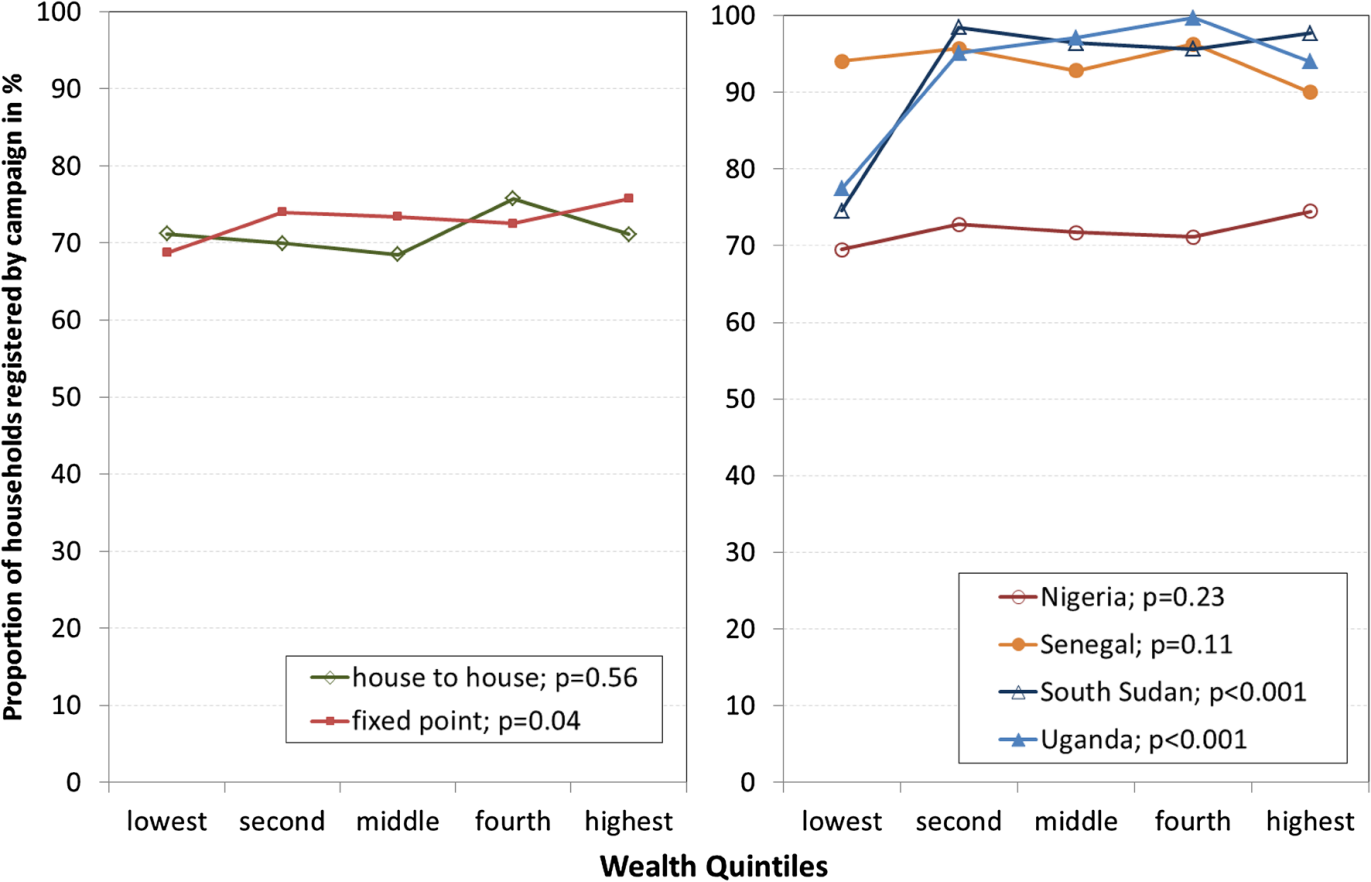
Registration in relation to wealth quintiles

Delivery strategy was not associated with registration rates nor with inequity in registration. House-to-house delivery campaigns were highly equitable (Fig. 3, left panel) with a concentration index of 0.005 (95 % CI: −0.002–0.01). However, campaigns that used fixed-point delivery appeared slightly more likely to register wealthier quintiles (concentration index of 0.018; 95 % CI: 0.003–034). Further examination of the campaigns with fixed-point delivery (Fig. 3, right panel) showed that not being able to register the poorest households was not a systematic flaw for campaigns that used fixed-point delivery, since it was only seen in two (South Sudan and Uganda) of the four campaigns.

#### Distribution strategy and registration rates

**Fig. 4:**
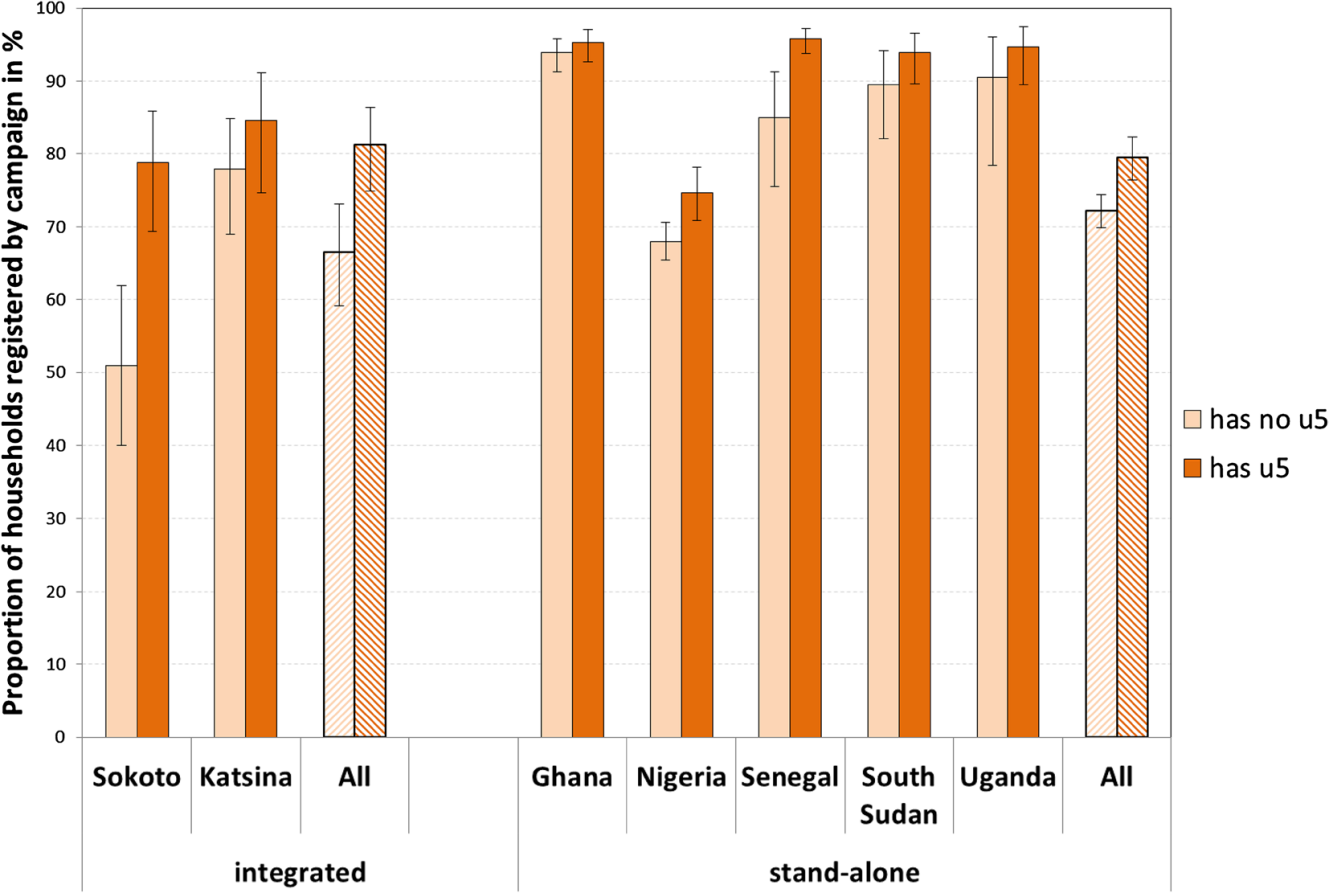
Comparison of household registration among integrated and stand-alone campaigns

Only two campaigns in the sample integrated ITN distributions with vaccination services (Sokoto and Katsina States, Nigeria). Katsina’s campaign achieved reasonably high registration rates (81 %) which was almost equal between households targeted by the vaccination (had children under five) and those not targeted (84 vs 78 %) (Fig. 4). In contrast, Sokoto’s registration rates were much lower overall and households with children under five were also more likely to be registered (78 vs 52 %) (Fig. 4). A comparison between integrated and stand-alone distribution was made by running a logistic regression model for only the Nigerian campaigns with registration as the dependent variable and distribution strategy as an independent variable along with covariates shown significant in the previous model (Table 3). This model gave a non-significant adjusted OR of 1.19 (95 % CI: −0.86–1.65) suggesting that distribution strategy was not associated with registration rates (data not shown).

#### Reasons for non-registration

**Fig. 5:**
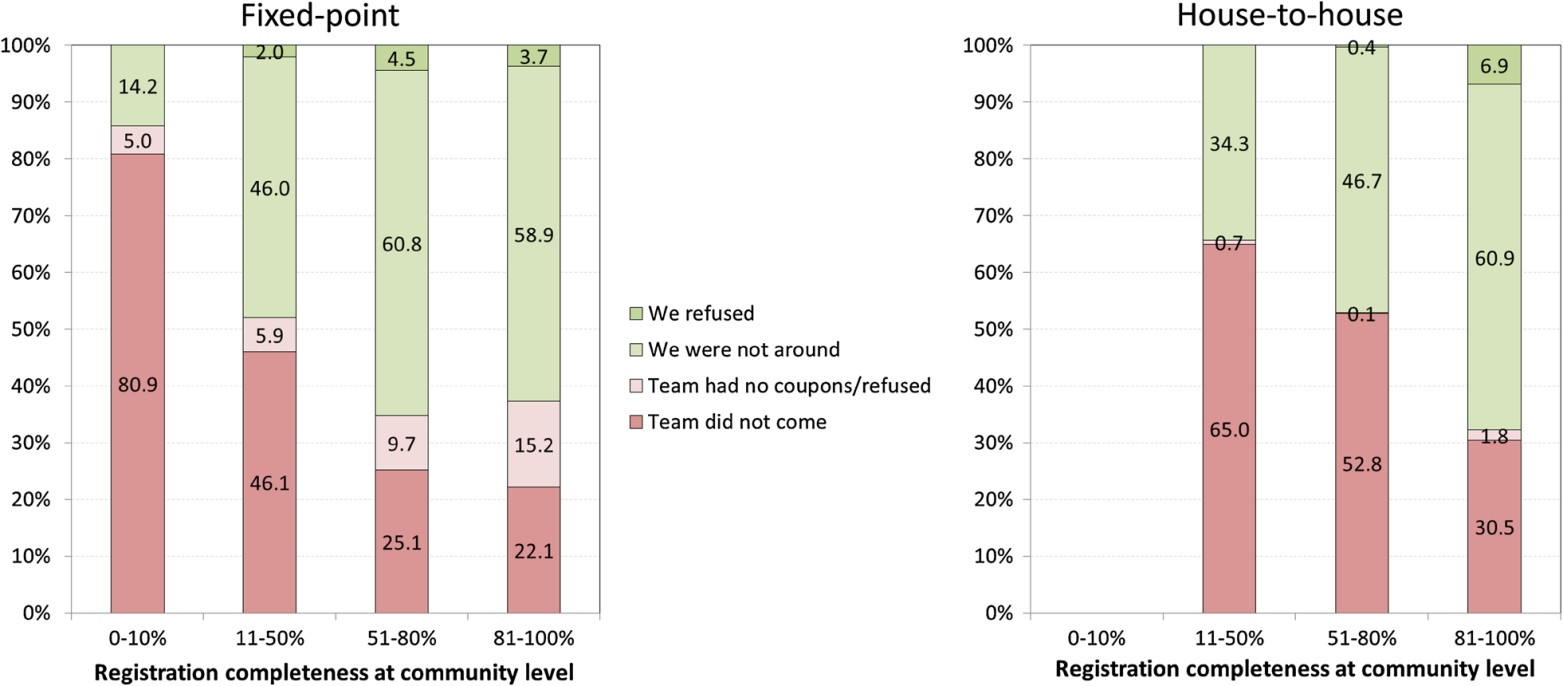
Reasons for non-registration by delivery strategy and community-level registration completeness

Figure 5 presents reasons for non-registration among 13,109 households by delivery strategy and by registration rates at the community level. The two main reasons were “the [registration] team did not come” and “we [the household members] were not around at that time” while “the team had no coupons or refused” and “we refused” were less common. Team-related reasons were clearly more common among households in villages with lower registration completeness. As community-level registration rates increased, household-related reasons for non-registration became increasingly more common while team-related reasons decreased. When community registration rates were above 80 %, over 60 % of households who were not registered said they refused or were not at home at the time. This trend was very similar regardless of the campaign’s delivery strategy. In a logistic regression model that adjusted for the surveys, the declining trend of team-related reasons with increasing community registration completeness was highly significant (p < 0.001) while the adjusted OR for house-to house delivery vs. fixed point of 1.14 was not significant (95 % CI: 0.45–2.78, p = 0.8). Neither wealth quintile nor household size were significantly associated with team-related reasons for non-registration.

## Discussion

### A successful registration process is critical for campaign success

In the surveys explored in this study, registration was highly associated with campaign effectiveness; campaigns that were less successful in registering households also resulted in the lower rates of household ownership of at least one ITN from the campaign. However, low registration rates did not necessarily compromise the next steps in the distribution process. Around 86 % of the households that were registered eventually received a net. This confirms that the ability of registration teams to reach households through door-to-door visits is the most important factor for the success of a campaign.

The importance of the registration process to campaign outcome has not been clearly demonstrated in the published literature so far. Although publications from Uganda [27] and Senegal [12] have examined campaign outcomes in these two countries, none have included registration as a survey outcome. A paper by Tokponnon et al. [19] presents results from mass distribution in Benin and compares the outcome in 12 districts. The association between registration completeness and campaign effectiveness was less clear as registration was high in all districts, varying between 80.3 and 96.7 % of sampled households. This is most probably due to household sampling occurring through the campaign listing, and therefore, households missed by the registration teams also were less likely to be included in the sample for the evaluation. This paper contributes to the literature by providing evidence that registration completeness is crucial for reaching households with a campaign net. Although the resulting odds ratio was uncommonly high, a strong association between registration and ownership is plausible since registration helps implementers identify households who need a net, pre-position appropriate quantities of ITNs at lower levels, and sensitize households on how and why it is important to participate in the mass campaign. Therefore, reducing barriers to household registration will increase the likelihood of a successful campaign.

Although differences in registration rates existed between the campaigns included in this study, they were seen across categories of delivery and net allocation strategies in the logistic regression models, suggesting that there are no systematic associations between campaign strategy and the proportion of households registered by the campaign. Similarly, results showed that integrated campaigns were not, in principle, more likely to result in more complete registration compared to stand-alone campaigns. This suggests that how well a campaign is organized will determine registration success, not necessarily its delivery, allocation, or distribution strategy.

The primary challenge identified in this analysis was reaching all households during the registration process. In Nigeria, where resulting coverage levels were lower, a closer examination revealed wide variations in registration rates within clusters. This probably reflects the difficulty in accessing hard-to-reach areas. The clusters that were almost completely missed by the registration team had few households. In fact, the most common reason for non-registration among communities with low registration rates was that the team did not come. On the other hand, among villages with higher registration rates, the absence of household members was more commonly mentioned as the reason for non-registration.

Larger household size and those with children under five were shown to be associated with increasing likelihood of registration. Although the data does not allow a determination as to the cause of this observation, one can hypothesize that these households were both better known to the registration staff and more likely to have at least one household member present at the day of the registration. The analysis also showed a consistently lower registration success in urban areas which most likely reflects urban residents’ higher mobility and lower willingness to cooperate in mass campaigns.

### Mass campaigns can rapidly scale up ownership, regardless of campaign strategy

All campaigns dramatically increased household ownership of at least one ITN. Pre-campaign household ownership levels ranged from 0.7 to 39.9 % and increased to 46.3–93.9 % post-campaign. This confirms that mass distribution campaigns can rapidly scale up household coverage where pre-campaign ownership is low, and this is true independent of the strategy used to distribute, deliver or allocate ITNs. The pre-campaign ownership levels presented in this paper included nets of any type as opposed to an ITN. At that time, ITNs were mainly given to pregnant women and children under five. Therefore, it is reasonable to assume that the pre-campaign ownership of an ITN was even lower.

### Choice of delivery strategy has little impact on ownership of at least one ITN

House-to-house delivery could be expected to be more successful than fixed-point as teams might have more flexibility in reaching specific households as issues such as households forgetting the date of the distribution or losing the net coupon can be avoided. However, the data from the 14 surveys does not suggest a significant association between delivery strategy and ownership of a net from the campaign. Instead, larger households were more likely to have at least one ITN while households with any children under five were less likely to obtain at least one ITN after controlling for having been registered. This finding appears contradictory at first glance as these households were found to be more likely to be registered. However, the results suggest that particularly small households with children were less likely to get an ITN once they were registered, compared to households of the same size without children. The data do not provide sufficient detail to determine why this might be, but it is possible that these are single-parent households with children that were too occupied with family matters to make it to the distribution. Further qualitative research might be useful to examine this question and determine whether special attention to this phenomenon may be in order.

### Choice of allocation strategy affects the proportion of households with enough ITNs

It is not surprising that fixed allocation campaigns did not achieve as high coverage of enough ITNs as UC allocation campaigns since the former did not attempt to achieve the UC target. However, comparing the two common UC allocation approaches (number of sleeping places or people in the household) to determine the number of nets needed did suggest that the sleeping place strategy had significant advantages over the allocation by number of household members. After adjusting for other factors, households from UC campaigns that used the sleeping place allocation approach were 60 % more likely to have enough ITNs compared to households that used a fixed allocation. This would imply that sleeping place counting is a more accurate method to define household needs, possibly by allowing more flexibility for families where the ratio of people per sleeping space is lower than 2.0. It could also be due to some households inflating their number of sleeping spaces to receive more nets. However, all the sleeping space campaigns (i.e., in Ghana, Senegal, and Uganda) in this analysis used a validation technique based on the number of household members in case households reported an unreliable number of sleeping spaces.

Overall, registration, access, and ownership of at least one ITN were shown to be quite equitable. Among households from UC campaigns, wealthier households initially appeared slightly more likely to have enough ITNs. This is probably because wealthier households tend to have fewer household members; however, when broken down further by type of allocation strategy, sleeping place allocation appeared to be more equitable than allocation by number of household members.

None of the UC allocation campaigns managed to provide exactly the right amount of ITNs to all households. Varying levels of oversupply were seen independent of the allocation strategy. Uganda, which used both approaches, for example, had the highest rates of oversupply. This suggests that differences were more likely due to campaign performance rather than choice of allocation strategy. Uganda’s excellent performance (in ownership of at least one ITN and having enough ITNs for all household members) was likely due to a significant oversupply of ITNs, rather than allocation strategy. Undersupply occurred primarily in larger households, suggesting that this may not be a systematic issue associated with allocation strategy but rather a problem of insufficient supply at the distribution point or house-to-house team, due to issues in the quantification, micro-planning, and issuing processes. Unfortunately, household surveys are not able to capture this aspect of campaigns and process evaluations will be needed to clarify this question.

### Study limitations

This analysis has limitations. Like any survey that relies on interviews with household respondents, these surveys were prone to potential recall and misclassification biases. Nonetheless, many aspects of demography such as proportion of children under five, currently pregnant women, and socio-economic characteristics for education and household assets were found to be as one would expect from other data sources, suggesting a high level of consistency. Furthermore, results were consistent in many ways within the dataset regarding trends with age and wealth quintiles and previously known net ownership so that in total, the results can be considered as being valid within the limits of the described range of precision.

Although the analysis included a large number of households, only 14 campaigns were analyzed and these were not systematically selected but rather opportunistically chosen from what was available. This meant that some analyses comparing the effectiveness of different strategies had small sample sizes. For example, only two of the 14 campaigns used a house-to-house delivery strategy Ghana and Cross River State (rural) in Nigeria] and two campaigns used the one ITN per two people allocation strategy (Cross River State in Nigeria and Uganda). Larger and more similar sample sizes between subgroups would better control for variation in implementation quality. However, it is very difficult to obtain such comprehensive data, as not all campaigns systematically evaluate outcomes with a survey robust enough to allow the kind of analysis presented here.

This is the first paper to synthesize the effect of mass campaign implementation strategies on ITN coverage outcomes across multiple countries. Although the results presented could contribute to making the best use of scarce resources, more research on the cost of various strategies is needed.

## Conclusion

This paper demonstrated some important facts to guide the decision-making process of a campaign strategy. All of the campaigns, irrespective of strategy, successfully increased ownership of at least one ITN. Delivery, distribution, or allocation strategy was not associated with receipt of at least one ITN from the campaign. Campaigns that used a universal coverage allocation, especially sleeping space allocation, were more effective in increasing the proportion of households with enough ITNs. The key determining factor for receipt of at least one ITN from the campaign was a successful registration process, which depends on the ability of community volunteers to reach households during the exercise. Maximizing registration completeness and using a universal coverage allocation are therefore likely to improve campaign outcomes.
